# Equilibrium-Based Finite Element Analysis of the Reissner–Mindlin Plate Bending Problem

**DOI:** 10.3390/ma18214969

**Published:** 2025-10-30

**Authors:** Zdzisław Więckowski, Paulina Świątkiewicz

**Affiliations:** 1 Independent Researcher, 90-001 Łódź, Poland; zdzislaw.wieckowski@p.lodz.pl; 2 Department of Structural Mechanics, Faculty of Civil Engineering, Architecture and Environmental Engineering, Lodz University of Technology, Al. Politechniki 6, 90-924 Łódź, Poland

**Keywords:** Reissner–Mindlin plate model, finite element method, equilibrium-based method, plate bending, a posteriori error estimation

## Abstract

A stress-based finite element approach to the Reissner–Mindlin plate bending problem is proposed. The rectangular Bogner–Fox–Schmit and triangular Hsieh–Clough–Tocher elements are applied to approximate the Southwell stress function describing the statically admissible stress field in a plate. To have some reference for the numerical results and estimate errors of the approximate solutions, two displacement-based elements with 12 and 22 degrees of freedom are also utilised. The variant of boundary conditions—known in the literature as 2D or hard BC—is analysed in the present study.

## 1. Introduction

The Reissner–Mindlin plate theory is pondered in numerous studies employing finite element methods. A thorough description of some elements, their assets and shortcomings may be found in [1,2]. Nevertheless, new approaches are still desirable to broaden the number of application possibilities and to overcome obstacles that limit some existing numerical methods.

In the case of the displacement-based elements that are used for moderately thick plates, only $C^{0}$ continuity is required. One of the most recognised problems that refers to Reissner–Mindlin theory is shear locking occurring as the plate thickness is reduced. A remedy for this obstacle is the technique of reduced or selective integration (e.g., [3,4]). Bathe and Dvorkin [5] introduced a different approach in the case of quadrilateral elements by special formulation of the element stiffness matrix. They interpolated transverse shear strains using shear strains evaluated at midpoints of element sides. Another interesting group of elements are those based on a “free formulation”. Bergan and Wang [6] derived a quadrilateral 12 DOF element in which transverse displacement is interpolated by polynomial shape functions, using complete polynomials of the second order degree to reproduce all rigid body and constant curvature modes in conjunction with a judiciously selected set of higher-order deformation functions. A similar approach was proposed by Liu et al. [7] in the case of their 16 DOF, 4-node element. Statics of a moderately thick plate and eigenvalue analysis were a concern of Buczkowski et al. [8], who proposed a 16-node locking-free element together with an adjusted foundation element based on the two-parameter Pasternak elastic foundation concept.

The importance of the Reissner–Mindlin plate theory for engineers and scientists is confirmed by the number of diverse numerical methods employed through decades in this research area. These are mixed formulations, e.g., based on the Hellinger–Reissner functional by Gruttmann and Wagner [9] or the one by Lee and Wong [10], the employing of the Hellinger–Reissner functional together with an isogeometric analysis with the use of NURBS shape functions by Kikis and Klinkel [11] or the one based on the modified Hu–Washizu functional by Batoz and Katili [12]. A finite volume method with boundary conditions fulfilled with the use of line boundary cells was implemented by Wheel [13]. We may also mention the boundary method with equilibrated basis functions, e.g., [14], plate elements for which matrices are obtained through analytical integration [15] and many others.

There also exists a variety of hybrid formulations that are based on equilibrium. Cook [16] developed hybrid 12 DOF quadrilateral elements based on triangular elements using the assumed stress hybrid approach. Maunder et al. [17] introduced a triangular hybrid plate element of general degree that exploits stress functions [18] to guarantee statically admissible moments and shear forces inside the element. Maunder and Izzuddin formulated a quadrilateral macro-element based on the assembly of triangular primitives [19]. Li et al. [20] proposed an 18 DOF triangular hybrid element based on a modified complementary energy principle. Different hybrid equilibrium formulations are described in [21]. Strong equilibrium in Reissner–Mindlin plates was also a matter of interest to Maunder et al. [22], who considered equilibrium recovery from conforming models.

The literature on the topic of Reissner–Mindlin plate elements is extensive and cannot be fully covered in a brief introduction. A detailed description of developments introduced since the 1960s may be found in Ref. [23].

This paper focuses on the equilibrium approach to the Reissner–Mindlin plate theory. It is an extension of the method shown by the authors in the analysis of thin plates [24]. In the present paper, the Southwell stress vector function [18] is employed in approximation of the bending and twisting moments together with the shear forces to guarantee static admissibility of the stress resultants. Components of the Southwell function are approximated with interpolation functions of the Bogner–Fox–Schmit element and the Hsieh–Clough–Tocher triangular macro-element [25]. The stress boundary conditions having the form of linear combinations of degrees of freedom are satisfied by the Lagrange multiplier method with the employment of linear multi-point constraint (MPC) elements [24,26]. In the present paper, the hard boundary conditions (see e.g., Refs. [27,28,29]) are considered, which means that the Kirchhoff kinematic condition is applied for the plate boundary.

The numerical results obtained by the proposed stress-based FE approach are compared with the outcomes gained with the help of the displacement-based FEM. In the latter approach, two locking-free elements with 12 and 22 degrees of freedom are employed. Having the two solutions—the stress- and displacement-based ones—the a posteriori error of the approximate solution is estimated by the Synge method [24,30].

This paper is organised as follows. The equations of the Reissner–Mindlin plate theory are briefly recalled in Section 2. The variational formulations in the stress and displacement formats are presented in the same section. Details related to the stress- and displacement-based finite element solutions are given in Section 3 and Section 4, respectively. Several examples of computations are given in Section 5. The paper is concluded in Section 6.

## 2. Formulation of the Reissner–Mindlin Plate Bending Problem

Let Ω and ∂Ω denote the region representing the plate mid-surface area and its boundary, respectively.

The equations describing the mechanical behaviour of the Reissner–Mindlin plate subjected to a static loading can be found, e.g., in [27,28,31].

**Figure 1:**
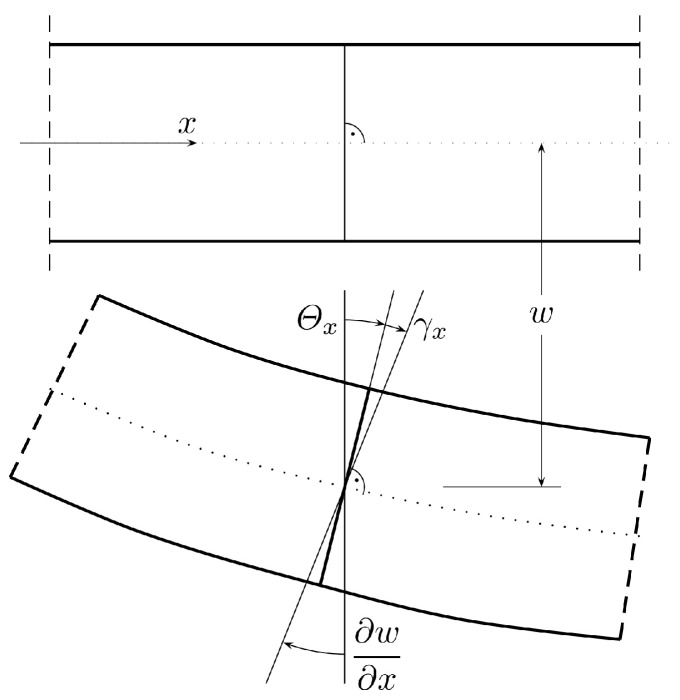
Plate deformation in the xz ($x_{1}x_{3}$) plane.

Geometric relations satisfied on region Ω (illustrated in Figure 1) take the following form:

(1) $$\begin{array}{cc} \kappa_{\alpha \beta }+\frac{1}{2}\;\left(\theta_{\alpha ,\beta }+\theta_{\beta ,\alpha }\right) & =0, \end{array}$$

(2) $$\begin{array}{cc} \gamma_{\alpha }-\left(w_{,\alpha }-\theta_{\alpha }\right) & =0 \end{array}$$

where α,β=1,2, symbol $\kappa_{\alpha \beta }$ denotes the curvatures, $\gamma_{\alpha }$ is the transverse shear strain in the $x_{\alpha }x_{3}$ plane and $\theta_{\alpha }$ means the rotation of the element normal to the plate mid-surface in the $x_{\alpha }x_{3}$ plane.

**Figure 2:**
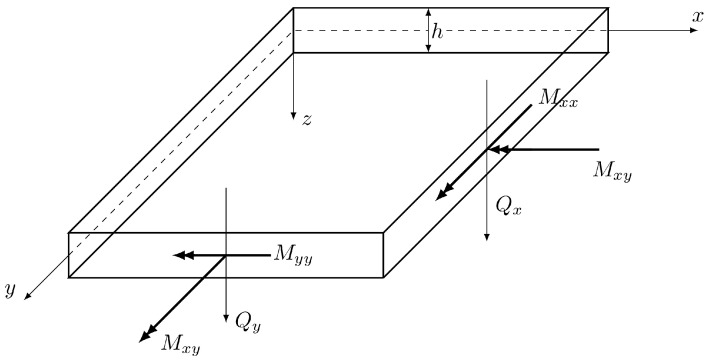
Stress resultants in a plate—sign convention.

The equilibrium of bending and twisting moments $M_{\alpha \beta }$ and shear forces $Q_{\alpha }$ (Figure 2), induced by a transverse distributed loading *q*, yields the following equations:

(3) $$\begin{array}{cc} M_{\alpha \beta ,\beta }-Q_{\alpha } & =0, \end{array}$$

(4) $$\begin{array}{cc} Q_{\alpha ,\alpha }+q & =0. \end{array}$$

The linear constitutive relation takes the following form:

(5) $$\begin{array}{cc} \kappa_{\alpha \beta } & =C_{\alpha \beta \gamma \delta }^{b}\;M_{\gamma \delta }, \end{array}$$

(6) $$\begin{array}{cc} \gamma_{\alpha } & =C_{\alpha \beta }^{s}\;Q_{\beta } \end{array}$$

where $C_{\alpha \beta \gamma \delta }^{b}$ indicates the compliance tensor related to bending deformation, whereas $C_{\alpha \beta }^{s}$ denotes the compliance tensor related to shear deformation. These two compliance tensors—for an isotropic material—can be expressed as follows:

(7) $$\begin{array}{c} C_{\alpha \beta \gamma \delta }^{b}=\frac{12}{E\;h^{3}}\;\left[\left(1+\nu \right)\;\delta_{\alpha \gamma }\;\delta_{\beta \delta }-\nu \;\delta_{\alpha \beta }\;\delta_{\gamma \delta }\right],\;C_{\alpha \beta }^{s}=\frac{1}{kGh}\;\delta_{\alpha \beta } \end{array}$$

where *E* and *G* are Young’s and Kirchhoff’s moduli, respectively, ν is Poisson’s ratio, *h* is the thickness of the plate, *k* means the shear correction factor and is set as k=5/6 in the case of homogeneous plates, and $\delta_{\alpha \beta }$ denotes Kronecker’s symbol.

The formulation of the considered boundary value problem must be completed with the boundary conditions. They can have a form of so-called “2D” or “hard” boundary conditions similar to those applied in the case of the Kirchhoff plate model or “3D” or “soft” boundary conditions usually utilised in the FE analyses of the Reissner–Mindlin plates. The conditions specified in Table 1—except for the hard conditions for the free edge—are described in Refs. [27,28,29,32].

Subscripts *n* and *s* used in Table 1 refer to the local co-ordinate system *n*–*s* introduced along the plate boundary, in which *n* is the normal axis, while *s* is the axis tangential to the edge.

In the present paper, the hard boundary conditions are considered with respect to the generalisation of them in the case of the free edge condition. A reason for such a choice of boundary conditions is explained in the further part of the section.

### 2.1. Construction of the Statically Admissible Stress Field

In the stress-based approach to the FEM, the Southwell stress function [33] is applied to satisfy the equilibrium equations (Equations (3) and (4)). The section forces, $M_{\alpha \beta }$ and $Q_{\alpha }$, are represented by the vector stress function $U_{\alpha }$, α=1,2,

(8) $$\begin{array}{cc} M_{\alpha \beta } & =\frac{1}{2}\;e_{\alpha \gamma }\;e_{\beta \delta }\;\left(U_{\gamma ,\delta }+U_{\delta ,\gamma }\right)-m_{0}\;\delta_{\alpha \beta }, \end{array}$$

(9) $$\begin{array}{cc} Q_{\alpha } & =\frac{1}{2}\;e_{\alpha \gamma }\;e_{\beta \delta }\;{\left(U_{\gamma ,\delta }+U_{\delta ,\gamma }\right)}_{,\beta }-m_{0,\alpha } \end{array}$$

where $e_{\alpha \gamma }$ is the permutation symbol, whereas function $m_{0}$ relates to surface loading as follows:

(10) $$m_{0,\alpha \alpha }=q.$$

Let us consider a local co-ordinate system *n*–*s* on boundary ∂Ω, where *n* and *s* denote axes normal and tangential to the boundary, respectively. The generalised boundary stress components can be expressed by the Southwell stress function

(11) $$\begin{array}{cc} M_{nn} & =U_{s,s}-m_{0}, \end{array}$$

(12) $$\begin{array}{cc} M_{ns} & =-\frac{1}{2}\;\left(U_{n,s}+U_{s,n}\right), \end{array}$$

(13) $$\begin{array}{cc} Q_{n} & =\frac{1}{2}\;\left(-U_{n,ss}+U_{s,ns}\right)-m_{0,n}. \end{array}$$

It is required to express the boundary conditions in terms of the stress function. To this end, the stress function components (or their derivatives) should be represented by the stress components. From Equation (11), the derivative of tangential component $U_{s}$ can be found easily

(14) $$U_{s,s}=M_{nn}+m_{0}.$$

Calculation of the derivative of Equation (12) with respect to *s* gives

(15) $$M_{ns,s}=-\frac{1}{2}\;\left(U_{n,ss}+U_{s,ns}\right).$$

Subtraction and addition of Equations (13) and (15) leads to the following pair of equations:

(16) $$\begin{array}{cc} -U_{n,ss} & =Q_{n}+M_{ns,s}+m_{0,n}, \end{array}$$

(17) $$\begin{array}{cc} U_{s,ns} & =Q_{n}-M_{ns,s}+m_{0,n}. \end{array}$$

Equation (16) shows the relation between the derivative of the normal component of the stress function and the Kirchhoff equivalent shearing force (e.g., Ref. [33])

(18) $$-U_{n,ss}=Q_{K}+m_{0,n}\;\mathrm{with}\;\;Q_{K}=Q_{n}+M_{ns,s}.$$

After inserting the derivative of Equation (14) with respect to variable *n* to Equation (17), we obtain the equation

(19) $$M_{nn,n}+m_{0,n}=Q_{n}-M_{ns,s}+m_{0,n}\;\mathrm{or}\;M_{nn,n}+M_{ns,s}-Q_{n}\equiv 0$$

which is identically satisfied due to the equilibrium equation (Equation (3)).

It follows from the above considerations that two stress boundary conditions are to be applied in the stress-based approach to the FEM. Using Equations (14) and (16), these conditions can be written in the form:

(20) $$\begin{array}{cc} U_{s,s}-m_{0}=M_{nn}=\bar{M}\; & \mathrm{on}\;\Gamma_{M}, \end{array}$$

(21) $$\begin{array}{cc} -U_{n,ss}-m_{0,n}=Q_{K}=\bar{Q}\; & \mathrm{on}\;\Gamma_{Q} \end{array}$$

where $\bar{M}$ and $\bar{Q}$ are given functions defined on boundary parts $\Gamma_{M}$ and $\Gamma_{Q}$, respectively.

The normal bending moment, $M_{nn}$, depends only on a derivative of one stress function component, $U_{s}$, whereas the Kirchhoff shear force depends only on a derivative of the other stress function component, $U_{n}$. Then, the hard boundary conditions can be easily enforced in the stress-based FE approach. Only these types of boundary conditions are considered in the present paper.

### 2.2. Stress-Based Variational Formulation of the Problem

The multiplication of Equations (1) and (2) by variations $\delta M_{\alpha \beta }$ and $\delta Q_{\alpha }$, respectively, integration of the result over the region Ω, and subsequent use of Green’s formula and the constitutive equations lead to the following equation:

(22) $$\begin{array}{c} \int_{\Omega }\left(C_{\alpha \beta \gamma \delta }^{b}\;M_{\gamma \delta }\;\delta \;M_{\alpha \beta }+C_{\alpha \beta }^{s}\;Q_{\beta }\;\delta Q_{\alpha }\right)\;dx=-\int_{\partial \Omega }\theta_{n}\;\delta \;M_{nn}\;ds+\int_{\partial \Omega }w\;\delta Q_{n}\;ds-\int_{\partial \Omega }\theta_{s}\;\delta \;M_{ns}\;ds\;\forall \;\left(\delta M_{\alpha \beta },\delta Q_{\alpha }\right). \end{array}$$

Before formulating the complementary work equation, let us consider the last two terms of the right-hand side of Equation (22). After using Equations (12) and (13), these two terms take the form:

(23) $$\begin{array}{cc} \int_{\partial \Omega }w\;\delta Q_{n}\;ds-\int_{\partial \Omega }\theta_{s}\;\delta \;M_{ns}\;ds= & \;\frac{1}{2}\;\int_{\partial \Omega }w\;\delta \left(-U_{n,ss}+U_{s,ns}\right)\;ds+\frac{1}{2}\;\int_{\partial \Omega }\theta_{s}\;\delta \left(U_{n,s}+U_{s,n}\right)\;ds=\\ \; & \;\frac{1}{2}\;\int_{\partial \Omega }w\;\delta {\left(U_{n,s}+U_{s,n}\right)}_{,s}\;ds-\int_{\partial \Omega }w\;\delta U_{n,ss}\;ds+\frac{1}{2}\;\int_{\partial \Omega }\theta_{s}\;\delta \left(U_{n,s}+U_{s,n}\right)\;ds=\\ \; & \;-\frac{1}{2}\;\int_{\partial \Omega }w_{,s}\;\delta \left(U_{n,s}+U_{s,n}\right)\;ds+\frac{1}{2}\;\int_{\partial \Omega }\theta_{s}\;\delta \left(U_{n,s}+U_{s,n}\right)\;ds-\int_{\partial \Omega }w\;\delta U_{n,ss}\;ds=\\ \; & \;-\frac{1}{2}\;\int_{\partial \Omega }\left(w_{,s}-\theta_{s}\right)\;\delta \left(U_{n,s}+U_{s,n}\right)\;ds-\int_{\partial \Omega }w\;\delta U_{n,ss}\;ds \end{array}$$

where integration by parts along the closed line, ∂Ω, and simple algebraic modifications are utilised. Using Equations (12), (16) and (23), we can express the right-hand-side of Equation (22) as follows:

(24) $$-\int_{\partial \Omega }\theta_{n}\;\delta \;M_{nn}\;ds+\int_{\partial \Omega }w\;\delta Q_{K}\;ds+\int_{\partial \Omega }\left(w_{,s}-\theta_{s}\right)\;\delta \;M_{ns}\;ds.$$

Then a variational formulation of the considered problem can be stated. Let the following set of statically admissible fields of section forces (generalised stresses) ($M_{\alpha \beta },Q_{\alpha }$), be defined:

(25) $$\begin{array}{cc} Y & =\left\{M_{\alpha \beta }\in H^{1}\left(\Omega \right),Q_{\alpha }\in L^{2}\left(\Omega \right):M_{\alpha \beta ,\beta }=Q_{\alpha }\;\mathrm{and}\;Q_{\alpha ,\alpha }=-q\;\mathrm{in}\;\Omega ,\;M_{nn}=\bar{M}\;\mathrm{and}\;Q_{K}=\bar{Q}\;\mathrm{on}\;\mathrm{part}\;\mathrm{of}\;\partial \Omega \right\}. \end{array}$$

The bending problem for the Reissner–Mindlin plate can be formulated under the principle of complementary work: find ($M_{\alpha \beta },Q_{\alpha })\in Y$ such that the following equation is satisfied:

(26) $$\begin{array}{c} \int_{\Omega }\left(C_{\alpha \beta \gamma \delta }^{b}\;M_{\gamma \delta }\;\delta \;M_{\alpha \beta }+C_{\alpha \beta }^{s}\;Q_{\beta }\;\delta Q_{\alpha }\right)\;dx=-\int_{\partial \Omega }\theta_{n}\;\delta \;M_{nn}\;ds+\int_{\partial \Omega }w\;\delta Q_{K}\;ds+\int_{\partial \Omega }\left(w_{,s}-\theta_{s}\right)\;\delta \;M_{ns}\;ds\\ \forall \;\left(\delta M_{\alpha \beta },\delta Q_{\alpha }\right)\in Y_{0} \end{array}$$

where

(27) $$\begin{array}{cc} Y_{0} & =\left\{M_{\alpha \beta }\in H^{1}\left(\Omega \right),Q_{\alpha }\in L^{2}\left(\Omega \right):M_{\alpha \beta ,\beta }=Q_{\alpha }\;\mathrm{and}\;Q_{\alpha ,\alpha }=0\;\mathrm{in}\;\Omega ,\;M_{nn}=0\;\mathrm{and}\;Q_{K}=0\;\mathrm{on}\;\mathrm{part}\;\mathrm{of}\;\partial \Omega \right\} \end{array}$$

denotes the space of statically admissible fields of generalised stresses. The last term in the integral along the plate boundary means that kinematic variables *w* and $\theta_{s}$ satisfy the equation

(28) $$w_{,s}-\theta_{s}=0\;\mathrm{on}\;\partial \Omega$$

as variation $\delta \;M_{ns}$ is arbitrary. This relation will be used further to construct a kinematically admissible displacement solution strictly corresponding to the statically admissible solution.

The equation of complementary work can be formulated in a more detailed form:

(29) $$\int_{\Omega }\left(M_{\alpha \beta }\;C_{\alpha \beta \gamma \delta }^{b}\;\delta \;M_{\gamma \delta }+Q_{\alpha }\;C_{\alpha \beta }^{s}\;\delta Q_{\beta }\right)\;dx+\int_{\Gamma_{\theta }}\bar{\theta }\;\delta \;M_{nn}\;ds-\int_{\Gamma_{w}}\bar{w}\;\delta Q_{K}\;ds=0\;\forall \;\left(\delta M_{\alpha \beta },Q_{\alpha }\right)\in Y_{0}$$

where $\bar{\theta }$ and $\bar{w}$ denote the prescribed functions given on boundary parts $\Gamma_{\theta }$ and $\Gamma_{w}$, respectively.

Formulation (29) is equivalent to the problem of minimising the functional of complementary energy on the set *Y*

(30) $$\begin{array}{c} \Sigma \left(M,Q\right)=\frac{1}{2}\;\int_{\Omega }\left(M_{\alpha \beta }\;C_{\alpha \beta \gamma \delta }^{b}\;M_{\gamma \delta }\;+Q_{\alpha }\;C_{\alpha \beta }^{s}\;Q_{\beta }\right)\;dx+\int_{\Gamma_{\theta }}\bar{\theta }\;M_{nn}\;ds-\int_{\Gamma_{w}}\bar{w}\;Q_{K}\;ds. \end{array}$$

### 2.3. Displacement-Based Formulation of the Problem

The variational formulation of the Reissner–Mindlin plate bending problem can be stated by multiplying equilibrium Equations (3) and (4) by $\left(-\delta \theta_{\alpha }\right)$ and δw, respectively, performing integration over area Ω, and then using the Green theorem. After employing the kinematic relations, the following equation can be written:

(31) $$\int_{\Omega }\left(M_{\alpha \beta }\;\delta \kappa_{\alpha \beta }+Q_{\alpha }\;\delta \gamma_{\alpha }\right)\;dx=\int_{\Omega }q\;\delta w\;dx-\int_{\partial \Omega }\left(M_{nn}\;\delta \theta_{n}+M_{ns}\;\delta \theta_{s}\right)\;ds+\int_{\partial \Omega }Q_{n}\;\delta w\;ds\;\forall \left(\delta \theta_{\alpha },\delta w\right).$$

After applying Equation (28), the component of the right-hand side of Equation (31) related to moment $M_{ns}$ becomes

(32) $$-\int_{\partial \Omega }M_{ns}\;\delta \theta_{s}\;ds=-\int_{\partial \Omega }M_{ns}\;\delta w_{,s}\;ds=\int_{\partial \Omega }M_{ns,s}\;\delta w\;ds$$

where integration by part along the closed line, ∂Ω, is utilised. Then, Equation (31) can be rewritten as follows:

(33) $$\begin{array}{c} \int_{\Omega }\left(M_{\alpha \beta }\;\delta \kappa_{\alpha \beta }+Q_{\alpha }\;\delta \gamma_{\alpha }\right)\;dx=\int_{\Omega }q\;\delta w\;dx+\int_{\partial \Omega }\left(-M_{nn}\;\delta \theta_{n}+Q_{K}\;\delta w\right)\;ds\\ \forall \left(\theta_{\alpha },w\right)\;\mathrm{such}\;\mathrm{that}\;w_{,s}-\theta_{s}=0\;\mathrm{on}\;\partial \Omega . \end{array}$$

Let us consider a space of kinematically admissible displacement field

(34) $$V=\{\left(\theta_{\alpha }\in H^{1}\left(\Omega \right),w\in H^{1}\left(\Omega \right)\right):\theta_{n}=\bar{\theta }\;\mathrm{on}\;\Gamma_{\theta },w=\bar{w}\;\mathrm{on}\;\Gamma_{w},w_{,s}-\theta_{s}=0\;\mathrm{on}\;\partial \Omega \}$$

where ${\bar{\theta }}_{\alpha }$ and $\bar{w}$ denote functions given on $\Gamma_{\theta }$ and $\Gamma_{w}$, respectively.

The bending problem for the Reissner–Mindlin plate can be formulated in displacements in the form of the virtual work equation: find $(\theta_{\alpha },w)\in V$ such that the following equation holds:

(35) $$\int_{\Omega }\left(D_{\alpha \beta \gamma \delta }^{b}\;\kappa_{\gamma \delta }\;\delta \kappa_{\alpha \beta }\;+D_{\alpha \beta }^{s}\;\gamma_{\beta }\;\delta \gamma_{\alpha }\right)\;dx=\int_{\Omega }q\;\delta w\;dx-\int_{\Gamma_{M}}\bar{M}\;\delta \theta_{n}\;ds+\int_{\Gamma_{Q}}\bar{Q}\;\delta w\;ds\;\forall \left(\delta \theta_{\alpha },\delta w\right)\in V_{0}$$

where

(36) $$V_{0}=\left\{\left(\theta_{\alpha }\in H^{1}\left(\Omega \right),w\in H^{1}\left(\Omega \right)\right):\theta_{n}=0\;\mathrm{on}\;\Gamma_{\theta },w=0\;\mathrm{on}\;\Gamma_{w},w_{,s}-\theta_{s}=0\;\mathrm{on}\;\partial \Omega \right\}$$

is the space of kinematically admissible displacement fields. $D_{\alpha \beta \gamma \delta }^{b}$ and $D_{\alpha \beta }^{s}$ are elasticity tensors inverse to the compliance tensors $C_{\alpha \beta \gamma \delta }^{b}$ and $C_{\alpha \beta }^{s}$, respectively, whereas $\bar{M}$ and $\bar{Q}$ are functions given on boundary parts $\Gamma_{M}$ and $\Gamma_{Q}$, respectively.

Equivalently, the problem considered can be formulated in terms of the minimisation problem: find field $(\theta_{\alpha },w)\in V$ minimising the functional of potential energy

(37) $$\Pi \left(\theta ,w\right)=\frac{1}{2}\int_{\Omega }\left(D_{\alpha \beta \gamma \delta }^{b}\;\kappa_{\gamma \delta }\;\kappa_{\alpha \beta }\;+D_{\alpha \beta }^{s}\;\gamma_{\beta }\;\gamma_{\alpha }\right)\;dx-\int_{\Omega }q\;w\;dx+\int_{\Gamma_{M}}\bar{M}\;\theta_{n}\;ds-\int_{\Gamma_{Q}}\bar{Q}\;w\;ds$$

on the set of kinematically admissible displacement fields, *V*.

## 3. Stress-Based FE Approximation

The plate stress resultants are expressed by the components of the Southwell stress function as shown in Equations (8) and (9). In the matrix notation, these equations can be written as follows:

(38) $$\sigma \equiv \left[\begin{array}{c} M_{xx}\\ M_{yy}\\ M_{xy}\\ Q_{x}\\ Q_{y} \end{array}\right]=\left[\begin{array}{c} \frac{\partial V}{\partial y}\\ \frac{\partial U}{\partial x}\\ -\frac{1}{2}\left(\frac{\partial U}{\partial y}+\frac{\partial V}{\partial x}\right)\\ \frac{1}{2}\left(-\frac{\partial^{2}U}{\partial y^{2}}+\frac{\partial^{2}V}{\partial x\partial y}\right)\\ \frac{1}{2}\left(\frac{\partial^{2}U}{\partial x\partial y}-\frac{\partial^{2}V}{\partial x^{2}}\right) \end{array}\right]-\left[\begin{array}{c} m_{0}\\ m_{0}\\ 0\\ \frac{\partial m_{0}}{\partial x}\\ \frac{\partial m_{0}}{\partial y} \end{array}\right]\equiv \left[\begin{array}{cc} 0 & \frac{\partial }{\partial y}\\ \frac{\partial }{\partial x} & 0\\ -\frac{1}{2}\;\frac{\partial }{\partial y} & -\frac{1}{2}\frac{\partial }{\partial x}\\ -\frac{1}{2}\;\frac{\partial^{2}}{\partial y^{2}} & \frac{1}{2}\;\frac{\partial^{2}}{\partial x\partial y}\\ \frac{1}{2}\;\frac{\partial^{2}}{\partial x\partial y} & -\frac{1}{2}\;\frac{\partial^{2}}{\partial x^{2}} \end{array}\right]\left[\begin{array}{c} U\\ V \end{array}\right]-M_{0}\equiv \nabla_{\mathrm{eqM}}\;U-M_{0}$$

where σ denotes the vector of the generalised stress and symbols x,y; and U,V are used for the Cartesian co-ordinates and stress function components.

**Figure 3:**
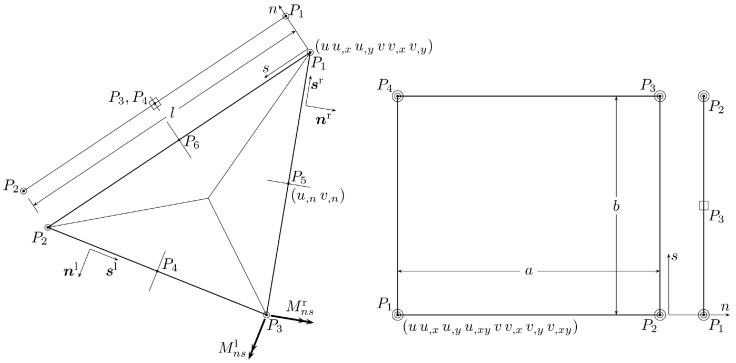
Elements used in the interpolation of the stress function. The triangular Hsieh–Clough–Tocher macro-element and the rectangular Bogner–Fox–Schmit element are shown on the left and right sides, respectively. Edge elements associated with the 2D elements are also presented.

In the Reissner–Mindlin plate theory, the shearing forces contribute to the strain energy. Then, they have to be square-integrable functions and are expressed by the second derivatives of the stress function. This means that the components of the Southwell function have to be interpolated with use of elements of class $C^{1}$. In the present paper, the triangular Hsieh–Clough–Tocher (HCT) element [25] and quadrilateral Bogner–Fox–Schmit (BFS) element [34] are employed. The elements are shown in Figure 3.

We can make a noticeable observation: In contrast to the Kirchhoff plate model where $C^{0}$ elements are applicable [24], we have to use $C^{1}$ elements for the Reissner–Mindlin plate model in the case of the stress-based approach for the FEM.

The HCT triangle has degrees of freedom representing the values of the stress function components *U* and *V*, their first derivatives with respect to *x* and *y* at the corners, and derivatives of *U* and *V* normal to the edges at the mid-side nodes of the triangle. In total, 24 degrees of freedom corresponding to the stress function are defined for the HCT element. Additionally, three other degrees of freedom—the Lagrange multipliers, described further—are introduced at corner nodes to fulfil equilibrium of point forces induced there by the twisting moment. In the case of the BFS rectangle, 32 degrees of freedom determine the stress function, and four auxiliary ones represent the Lagrange multipliers. All of them are defined at the element corners. The vectors of degrees of freedom for the above two elements have the forms:

(39) $$\begin{array}{cc} a_{\mathrm{HCT}} & ={\left[\begin{array}{ccccccccccccccccccc} u_{1} & u_{1,x} & u_{1,y} & v_{1} & v_{1,x} & v_{1,y} & \Lambda_{1} & u_{2} & \ldots & \Lambda_{2} & u_{3} & \ldots & \Lambda_{3} & u_{4,n} & v_{4,n} & u_{5,n} & v_{5,n} & u_{6,n} & v_{6,n} \end{array}\right]}^{T}, \end{array}$$

(40) $$\begin{array}{cc} a_{\mathrm{BFS}} & ={\left[\begin{array}{cccccccccccccccccc} u_{1} & u_{1,x} & u_{1,y} & u_{1,xy} & v_{1} & v_{1,x} & v_{1,y} & v_{1,xy} & \Lambda_{1} & u_{2} & \ldots & \Lambda_{2} & u_{3} & \ldots & \Lambda_{3} & u_{4} & \ldots & \Lambda_{4} \end{array}\right]}^{T} \end{array}$$

where some terms are not listed for brevity, and commas indicate the derivatives with respect to co-ordinates *x* or *y* and the directions normal to element edges in the case of mid-size nodes.

The components of the Southwell stress function are approximated by the use of the shape functions matrix N

(41) $$\begin{array}{cc} \left[\begin{array}{c} U\\ V \end{array}\right] & \equiv U=N\;a. \end{array}$$

Inserting the last equation to Equation (38) leads to the following relation:

(42) $$\begin{array}{cc} \sigma & =\nabla_{\mathrm{eqM}}\;N\;a-M_{0}\equiv B\;a-M_{0} \end{array}$$

where, for example, matrix B is shown in the case of the HCT element

(43) $$\begin{array}{cc} B & =\frac{1}{2}\;\left[\begin{array}{ccccccccccccc} 0 & 0 & 0 & 2N_{1,y} & 2N_{2,y} & 2N_{3,y} & 0 & \ldots & \ldots & 0 & 2N_{10,y} & \ldots & \ldots \\ 2N_{1,x} & 2N_{2,x} & 2N_{3,x} & 0 & 0 & 0 & 0 & \ldots & \ldots & 2N_{10,x} & 0 & \ldots & \ldots \\ -N_{1,y} & -N_{2,y} & -N_{3,y} & -N_{1,x} & -N_{2,x} & -N_{3,x} & 0 & \ldots & \ldots & -N_{10,y} & -N_{10,x} & \ldots & \ldots \\ -N_{1,yy} & -N_{2,yy} & -N_{3,yy} & N_{1,xy} & N_{2,xy} & N_{3,xy} & 0 & \ldots & \ldots & -N_{10,yy} & -N_{10,xy} & \ldots & \ldots \\ N_{1,xy} & N_{2,xy} & N_{3,xy} & -N_{1,xx} & -N_{2,xx} & -N_{3,xx} & 0 & \ldots & \ldots & -N_{10,xy} & -N_{10,xx} & \ldots & \ldots \end{array}\right]. \end{array}$$

In Equation (43), only blocks of columns related to nodes no. 1 and 4 are shown for brevity. After inserting Equation (43) into Equation (29), the equation of complementary work takes the form:

(44) $$\delta a^{T}\left(\int_{\Omega }B^{T}\;C\;\left(B\;a-M_{0}\right)\;dx-f\left(\bar{w},\bar{\theta }\right)\right)=0\;\forall \delta a\;\mathrm{such}\;\mathrm{that}\;B\delta a\in Y_{0}$$

where C denotes the compliance matrix, which has either of the forms

(45) $$C=\left[\begin{array}{ccccc} \frac{1}{D} & -\frac{\nu }{D} & 0 & 0 & 0\\ -\frac{\nu }{D} & \frac{1}{D} & 0 & 0 & 0\\ 0 & 0 & \frac{2\;(1+\nu )}{D} & 0 & 0\\ 0 & 0 & 0 & \frac{1}{S} & 0\\ 0 & 0 & 0 & 0 & \frac{1}{S} \end{array}\right],\;C=\left[\begin{array}{ccccc} C_{x} & C_{1} & 0 & 0 & 0\\ C_{1} & C_{y} & 0 & 0 & 0\\ 0 & 0 & C_{xy} & 0 & 0\\ 0 & 0 & 0 & C_{x}^{s} & 0\\ 0 & 0 & 0 & 0 & C_{y}^{s} \end{array}\right]$$

in the case of an isotropic or orthotropic plate, respectively, while term $\delta a^{T}\;f\left(\bar{w},\bar{\theta }\right)$ represents the linear term in Equation (29). In Equation (45), $D=Eh^{3}/12$, S=kGh, and compliance constants $C_{x}$, $C_{y}$, $C_{1}$, $C_{xy}$ correspond to the bending deformation while $C_{x}^{s}$, $C_{y}^{s}$ correspond to the shear deformation of the plate. Equation (44) is equivalent to the following linear set of algebraic equations

(46) $$\begin{array}{cc} \; & K\;a=F\;\mathrm{with}\;\\ \; & K=\int_{\Omega }B^{T}\;C\;B\;dx,\;F=\int_{\Omega }B^{T}\;C\;M_{0}\;dx+f\left(\bar{w},\bar{\theta }\right). \end{array}$$

The specific solution of the equilibrium equations, function $m_{0}$, satisfying Equation (10) and corresponding to a uniformly distributed load $q\left(x,y\right)=q_{0}=\mathrm{const}$, is assumed in the form:

(47) $$m_{0}\left(x,y\right)=\frac{q_{0}}{4}\;\left({\left(x-x_{0}\right)}^{2}+{\left(y-y_{0}\right)}^{2}\right)\;\;\mathrm{with}\;x_{0},y_{0}=\mathrm{const}.$$

Equation (10) can be solved numerically, but in such a case, the equilibrium Equations (3) and (4) are not exactly fulfilled.

### 3.1. Equilibrium of Corner Nodes

The twisting moments acting on edges of an element crossing at a corner are linked with the point force acting at this corner. In the case of any internal node (not positioned on boundary ∂Ω), the forces related to corners of surrounding elements are in equilibrium. When the components of the Southwell stress function are of $C^{1}$ class, these forces are also in equilibrium for nodes placed inside straight parts of the boundary. This notice follows from the continuity of the twisting moment represented by the derivatives of the stress function components. The equilibrium for the forces acting at the corner nodes placed on the plate boundary is enforced by the use of the Lagrange multiplier method using the way that is described in Ref. [24].

The Lagrange multiplier technique is implemented by the modification of the compliance element matrix by adding as many rows and columns as the number of corners the element has. These additional rows and columns contain the terms defined below. Let us consider the following notation:

(48) $$\begin{array}{cc} c_{1} & =n_{1}^{r}\;n_{2}^{r}-n_{1}^{l}\;n_{2}^{l}, \end{array}$$

(49) $$\begin{array}{cc} c_{2} & =\frac{1}{2}\;\left({\left(n_{1}^{r}\right)}^{2}-{\left(n_{2}^{r}\right)}^{2}-\left({\left(n_{1}^{l}\right)}^{2}-{\left(n_{2}^{l}\right)}^{2}\right)\right) \end{array}$$

where $n_{\alpha }^{r}$, $n_{\alpha }^{l}$ (α=1,2) are components of the vectors normal to edges crossing at the common corner (see Figure 3). Non-zero terms related to the appropriate Lagrange multipliers are localised in the matrix blocks lying along the diagonal of the element matrix. The single blocks for the HCT (left) and BFS (right) elements are shown below

(50) $$\begin{array}{ccccccc} ⋆ & \cdot & \cdot & \cdot & \cdot & \cdot & 0\\ \cdot & ⋆ & \cdot & \cdot & \cdot & \cdot & c_{1}\\ \cdot & \cdot & ⋆ & \cdot & \cdot & \cdot & -c_{2}\\ \cdot & \cdot & \cdot & ⋆ & \cdot & \cdot & 0\\ \cdot & \cdot & \cdot & \cdot & ⋆ & \cdot & -c_{2}\\ \cdot & \cdot & \cdot & \cdot & \cdot & ⋆ & -c_{1}\\ 0 & c_{1} & -c_{2} & 0 & -c_{2} & -c_{1} & 0 \end{array}\;\;\mathrm{or}\;\;\begin{array}{ccccccccc} ⋆ & \cdot & \cdot & \cdot & \cdot & \cdot & \cdot & \cdot & 0\\ \cdot & ⋆ & \cdot & \cdot & \cdot & \cdot & \cdot & \cdot & 0\\ \cdot & \cdot & ⋆ & \cdot & \cdot & \cdot & \cdot & \cdot & c_{3}\\ \cdot & \cdot & \cdot & ⋆ & \cdot & \cdot & \cdot & \cdot & 0\\ \cdot & \cdot & \cdot & \cdot & ⋆ & \cdot & \cdot & \cdot & 0\\ \cdot & \cdot & \cdot & \cdot & \cdot & ⋆ & \cdot & \cdot & c_{3}\\ \cdot & \cdot & \cdot & \cdot & \cdot & \cdot & ⋆ & \cdot & 0\\ \cdot & \cdot & \cdot & \cdot & \cdot & \cdot & \cdot & ⋆ & 0\\ 0 & 0 & c_{3} & 0 & 0 & c_{3} & 0 & 0 & 0 \end{array},$$

where stars show the positions of the diagonal elements of the compliance matrix; $c_{3}=1$ in the case of corners $P_{1}$ and $P_{3}$ or $c_{3}=-1$ in the case of corners $P_{2}$ and $P_{4}$. The corners numbering is shown in Figure 3.

### 3.2. Enforcing the Boundary Conditions

The stress boundary conditions (20) and (21) have the form of linear constraints for the degress of freedom defining the Southwell stress function. These conditions are fulfilled by the use of the Lagrange multiplier method with the help of the multi-point constraints (MPCs) element concept described in Refs. [24,26]. The MPC (edge) elements are illustrated in Figure 3.

In the case of elements considered in the present paper, the stress function is approximated with polynomials of the third degree, which means that the normal bending moment, $M_{nn}$, and the Kirchhoff shear force are represented by the quadratic and linear functions, respectively. The cubic interpolation functions allow us to satisfy the equilibrium equations on the plate boundary in the case of uniform surface loading *q*. The resultant forces acting on the plate boundary can be represented in the the following forms:

(51) $$\begin{array}{cc} M_{nn}=n_{1}^{2}\;M_{11}+n_{2}^{2}\;M_{22}+2\;n_{1}\;n_{2}\;M_{12} & =B_{M}\;a-m_{0}, \end{array}$$

(52) $$\begin{array}{cc} Q_{K}=n_{1}\;Q_{1}+n_{2}\;Q_{2}+\left(-n_{2}\;\frac{\partial }{\partial x_{1}}+n_{1}\;\frac{\partial }{\partial x_{2}}\right)\;\left(n_{1}n_{2}\;\left(M_{22}-M_{11}\right)+\left(n_{1}^{2}-n_{2}^{2}\right)\;M_{12}\right)-m_{0},n\; & =B_{Q}\;a-m_{0,n} \end{array}$$

and the boundary conditions for an edge element can be written as follows:

(53) $$\begin{array}{cc} f_{M}\left(s\right)\equiv B_{M}\left(s\right)\;a^{e}-\left(a_{2}\;s^{2}+a_{1}\;s+a_{0}\right)-\left(A_{2}\;s^{2}+A_{1}\;s+A_{0}\right)=0\; & \forall s\in (0,l), \end{array}$$

(54) $$\begin{array}{cc} f_{Q}\left(s\right)\equiv B_{Q}\left(s\right)\;a^{e}-\left(b_{1}\;s+b_{0}\right)-\left(B_{1}\;s+B_{0}\right)=0\; & \forall s\in (0,l) \end{array}$$

where $a_{k},k=0,1,2$ and $b_{k},k=0,1$ are coefficients of polynomials representing the distribution of function $m_{0}$ and its normal derivative, respectively, whereas $A_{k},k=0,1,2$ and $B_{k},k=0,1$ are coefficients of polynomials representing functions $\bar{M}$ and $\bar{Q}$, respectively. In the above two equations, vector $a^{e}$ contains the degrees of freedom linked with the specific edge segment (MPC element).

The constraints for elements of vector $a^{e}$ can be found by writing three equations for function $f_{M}$

(55) $$\begin{array}{c} f_{M}\left(0\right)=0,\;f_{M}\left(l\right)=0,\;\frac{1}{2}\;\frac{df_{M}}{ds}\left(0\right)-\frac{3}{l^{2}}\;\left(f_{M}\left(0\right)+f_{M}\left(l\right)\right)=0 \end{array}$$

and two equations for function $f_{Q}$

(56) $$\begin{array}{c} f_{Q}\left(0\right)=0,\;\frac{1}{2}\;\frac{df_{Q}}{ds}\left(0\right)=0. \end{array}$$

In the case of the HCT triangle, the linear constraints related to the boundary conditions have the following matrix form:

(57) $$\begin{array}{c} \left[\begin{array}{ccccccccccccccc} 0 & s^{2} & -sc & 0 & -sc & c^{2} & 0 & 0 & 0 & 0 & 0 & 0 & 0 & 0 & \\ 0 & 0 & 0 & 0 & 0 & 0 & 0 & 0 & s^{2} & -sc & 0 & -sc & c^{2} & 0 & \\ -\frac{6s}{l^{3}} & 0 & 0 & \frac{6c}{l^{3}} & 0 & 0 & 0 & \frac{6s}{l^{3}} & 0 & 0 & -\frac{6c}{l^{3}} & 0 & 0 & 0 & 0_{5\times 7}\\ -\frac{12c}{l^{3}} & \frac{6sc}{l^{2}} & -\frac{6c^{2}}{l^{2}} & -\frac{12s}{l^{3}} & \frac{6s^{2}}{l^{2}} & -\frac{6sc}{l^{2}} & 0 & \frac{12c}{l^{3}} & \frac{6sc}{l^{2}} & -\frac{6c^{2}}{l^{2}} & \frac{12s}{l^{3}} & \frac{6s^{2}}{l^{2}} & -\frac{6sc}{l^{2}} & 0 & \\ \frac{6c}{l^{2}} & -\frac{4sc}{l} & \frac{4c^{2}}{l} & \frac{6s}{l^{2}} & -\frac{4s^{2}}{l} & \frac{4sc}{l} & 0 & -\frac{6c}{l^{2}} & -\frac{2sc}{l} & \frac{2c^{2}}{l} & -\frac{6s}{l^{2}} & -\frac{2s^{2}}{l} & \frac{2sc}{l} & 0 \end{array}\right]\;a^{e}=\\ \left[\begin{array}{c} {\bar{a}}_{0}\\ {\bar{a}}_{2}\;l^{2}+{\bar{a}}_{1}l+{\bar{a}}_{0}\\ \left(-2{\bar{a}}_{2}\;l^{2}-3{\bar{a}}_{1}l-6{\bar{a}}_{0}\right)/l^{2}\\ {\bar{b}}_{1}\\ {\bar{b}}_{0} \end{array}\right] \end{array}$$

where ${\bar{a}}_{k}=a_{k}+A_{k},\;k=0,1,2$ and ${\bar{b}}_{k}=b_{k}+B_{k},\;k=0,1$, $c=n_{1}$, $s=n_{2}$; *l* is the length of the edge of the element adjacent to the boundary (shown in Figure 3), and the vector of degrees of freedom for the MPC element is defined as follows:

(58) $$a^{e}={\left[\begin{array}{ccccccccccccccccccccc} u_{1} & u_{1,x} & u_{1,y} & v_{1} & v_{1,x} & v_{1,y} & \Lambda_{1} & u_{2} & u_{2,x} & u_{2,y} & v_{2} & v_{2,x} & v_{2,y} & \Lambda_{2} & u_{3,n} & v_{3,n} & \lambda_{1} & \lambda_{2} & \lambda_{3} & \lambda_{4} & \lambda_{5} \end{array}\right]}^{T}.$$

After comparing the first two equations in Equation (57), one can notice that, for the neighboring edge elements, the second equation of the specific element and the first equation of the next element (with respect to the direction of axis *s*) are the same. Then, to avoid singularity of the matrix of the global finite element equations system, one of the Lagrange multipliers defined in the common node should be set to zero. Having the matrix and the vector given on the left-hand side and right-hand side of Equation (58), respectively, it is easy to define the MPC element matrix and vector using the procedure described in detail in Refs. [24,26].

The linear constraints related to the stress boundary conditions in the case of the BFS rectangle are not specified in the paper as they can be derived in the same manner as shown above.

## 4. Displacement-Based FE Approximation

**Figure 4:**
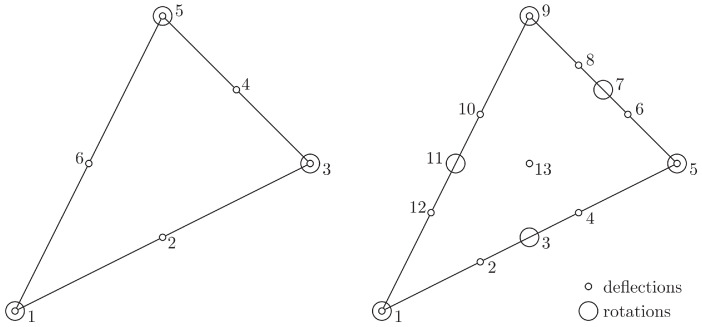
Displacement-based elements: the six-node, 12 dof element DM12 (**left**) and the thirteen-node, 22 dof element DM22 (**right**).

Two triangular elements are employed to approximate the displacement and rotation fields within the displacement-based FEM framework. To prevent shear locking, interpolation functions are chosen such that the polynomials approximating the rotation fields and derivatives of the deflection field are of the same degree. In the first element (DM12), deflections are approximated by quadratic polynomials, while rotations are approximated by linear ones. In the more accurate element (DM22), cubic and quadratic polynomials are used for deflections and rotations, respectively. The two elements are illustrated in Figure 4.

For the DM12 element, the relation between the generalised displacement field, u, and the element degrees of freedom has the following matrix form:

(59) $$u\equiv \left[\begin{array}{c} w\\ \Theta_{x}\\ \Theta_{y} \end{array}\right]=\left[\begin{array}{cccc} N_{1} & 0 & 0 & N_{2}\\ 0 & {\tilde{N}}_{1} & 0 & 0\\ 0 & 0 & {\tilde{N}}_{1} & 0 \end{array}\right|\left.\begin{array}{cccc} N_{3} & 0 & 0 & N_{4}\\ 0 & {\tilde{N}}_{3} & 0 & 0\\ 0 & 0 & {\tilde{N}}_{3} & 0 \end{array}\right|\left.\begin{array}{cccc} N_{5} & 0 & 0 & N_{6}\\ 0 & {\tilde{N}}_{5} & 0 & 0\\ 0 & 0 & {\tilde{N}}_{5} & 0 \end{array}\right]\;a^{e}$$

where $N_{i}$ (i=1,…,6) and ${\tilde{N}}_{i}$ (i=1,3,5) are the interpolation functions corresponding to quadratic and linear polynomials, respectively; and $a^{e}$ denotes the element degrees of freedom vector

(60) $$\begin{array}{cc} a^{e} & ={\left[\begin{array}{cccccccccccc} w_{1} & \Theta_{x1} & \Theta_{y1} & w_{2} & w_{3} & \Theta_{x3} & \Theta_{y3} & w_{4} & w_{5} & \Theta_{x5} & \Theta_{y5} & w_{6} \end{array}\right]}^{T} \end{array}$$

For DM22, the counterparts of Equations (59) and (60) are given by

(61) $$u\equiv \left[\begin{array}{c} w\\ \Theta_{x}\\ \Theta_{y} \end{array}\right]=\left[\begin{array}{ccccccc} N_{1} & 0 & 0 & N_{2} & 0 & 0 & N_{4}\\ 0 & {\tilde{N}}_{1} & 0 & 0 & {\tilde{N}}_{3} & 0 & 0\\ 0 & 0 & {\tilde{N}}_{1} & 0 & 0 & {\tilde{N}}_{3} & 0 \end{array}\right|\left.\begin{array}{cccc} N_{5} & 0 & 0 & \ldots \\ 0 & {\tilde{N}}_{5} & 0 & \ldots \\ 0 & 0 & {\tilde{N}}_{5} & \ldots \end{array}\right|\left.\begin{array}{cccc} N_{9} & 0 & 0 & \ldots \\ 0 & {\tilde{N}}_{9} & 0 & \ldots \\ 0 & 0 & {\tilde{N}}_{9} & \ldots \end{array}\right|\left.\begin{array}{c} N_{13}\\ 0\\ 0 \end{array}\right]\;a^{e}$$

and

(62) $$\begin{array}{cc} a^{e} & ={\left[\begin{array}{ccccccccccccc} w_{1} & \Theta_{x1} & \Theta_{y1} & w_{2} & \Theta_{x3} & \Theta_{y3} & w_{4} & w_{5} & \Theta_{x5} & \Theta_{y5} & w_{6} & \ldots w_{12} & w_{13} \end{array}\right]}^{T} \end{array}$$

where $N_{i}$ (i=1,2,4,5,6,8,9,10,12,13) and ${\tilde{N}}_{i}$ (i=1,3,5,7,9,11) are the interpolation functions corresponding to cubic and quadratic polynomials, respectively.

When hard boundary conditions are imposed, the nodal degrees of freedom on boundary nodes are constrained by the Kirchhoff kinematic condition, $w_{,s}-\theta_{s}=0$. For each element’s edge lying on the plate boundary, this condition reduces to a polynomial (in variable *s*) of the first or second degree for DM12 or DM22, respectively. Since all the coefficients in these polynomials vanish, the following linear algebraic system has to be satisfied for edge 1–3 of element DM12 (see Figure 4):

(63) $$\left[\begin{array}{ccccccc} \frac{4}{l^{2}} & -\frac{s}{l} & \frac{c}{l} & -\frac{8}{l^{2}} & \frac{4}{l^{2}} & \frac{s}{l} & -\frac{c}{l}\\ -\frac{3}{l} & s & -c & \frac{4}{l} & -\frac{1}{l} & 0 & 0 \end{array}\right]\left[\begin{array}{c} w_{1}\\ \Theta_{1x}\\ \Theta_{1y}\\ w_{2}\\ w_{3}\\ \Theta_{3x}\\ \Theta_{3y} \end{array}\right]=0$$

and for edge 1–5 of element DM22

(64) $$\left[\begin{array}{cccccccccc} -\frac{27}{l} & 4\;\frac{s}{l^{2}} & -4\;\frac{c}{l^{2}} & \frac{81}{l^{3}} & -8\;\frac{s}{l^{2}} & 8\;\frac{c}{l^{2}} & -\frac{81}{l^{3}} & {\frac{27}{l}}^{3} & 4\;\frac{s}{l^{2}} & -4\;\frac{c}{l^{2}}\\ \frac{18}{l^{2}} & -3\;\frac{s}{l} & 3\;\frac{c}{l} & -\frac{45}{l^{2}} & 4\;\frac{s}{l} & -4\;\frac{c}{l} & \frac{36}{l^{2}} & -\frac{9}{l^{2}} & -\frac{s}{l} & \frac{c}{l}\\ -\frac{11}{2l} & s & -c & \frac{9}{l} & 0 & 0 & -\frac{9}{2l} & \frac{1}{l} & 0 & 0 \end{array}\right]\left[\begin{array}{c} w_{1}\\ \Theta_{1x}\\ \Theta_{1y}\\ w_{2}\\ \Theta_{3x}\\ \Theta_{3y}\\ w_{4}\\ w_{5}\\ \Theta_{5x}\\ \Theta_{5y} \end{array}\right]=0$$

In Equations (63) and (64), the symbols *c*, *s* and *l* have the same meaning as in Equation (57). These constraints are enforced by the Lagrange multiplier method, introducing appropriate MPC elements as in the stress-based formulation.

## 5. Numerical Examples

The following section presents examples of calculations performed for various boundary conditions and plate thickness values. The results obtained using the equilibrium-based elements (HCT and BFS) are compared with analytic solutions, the results found in the literature (for some other finite elements), and the results obtained by a triangular displacement-based elements with 12 and 22 degrees of freedom (DM12 and DM22, respectively).

### 5.1. Uniformly Loaded Square Plates

**Figure 5:**
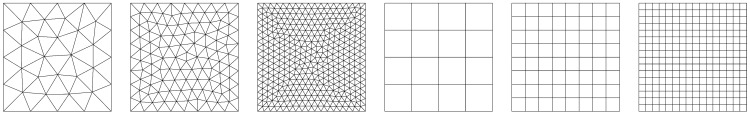
Element grids used in computations.

The first example is a square plate subjected to a uniformly distributed load *q*. The side length of the plate is a=10. Material properties used in calculations are E=10.92 and ν=0.3, as in Reference [35], which gives the plate stiffness $Eh^{3}/\left(12\left(1-\nu^{2}\right)\right)$ of $h^{3}$. Two values of the plate thickness are considered, h=0.1 and h=1.0, to show good performance of the elements in the case of thin and moderately thick plates. The intensity of the uniform loading *q* is 1.0. Element grids of various density, shown in Figure 5, are employed in the calculations. The maximum element size $h_{\max}$ is taken as a/4, a/8 and a/16.

**Figure 6:**
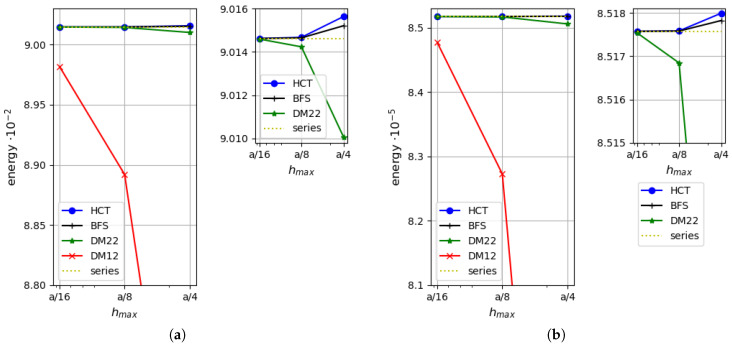
Strain energy for a plate: (**a**) for h=1.0; (**b**) for h=0.1.

First, a simply supported case with hard boundary conditions is considered. Convergence of two equilibrium-based elements (HCT and BFS) and two displacement-based elements (DM12 and DM22) are verified. The results are compared with the analytical solutions for the thin plate limit [31] and for thick plates with consideration of the shear deformation (in series form) [35]. Figure 6 presents a comparison of the strain energy for the analysed plate depending on the element size. Two plate thicknesses are considered: h=1.0 (on the left side of the figure) and h=0.1 (on the right side). It should be noted that by means of the stress-based method, the upper bound for the strain energy is found, whereas the kinematically admissible approach gives the lower bound. All the solutions are monotonically convergent to the exact values of the strain energy. The convergence rate of the displacement-based elements is visibly slower in comparison with the stress-based ones (even the more accurate one—DM22). The diagram corroborates that the statically admissible solutions surpass the kinematically admissible ones for both plate thicknesses. When thin plates are analysed, the results converge to the solution of Kirchhoff’s thin plate [31].

**Figure 7:**
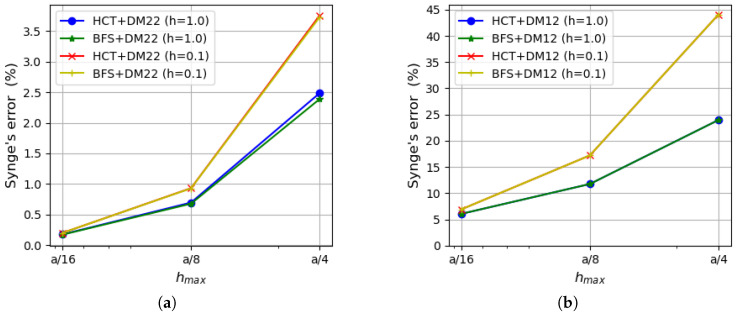
Relative error evaluated by Synge’s method: (**a**) using the DM22 element and stress-based elements; (**b**) using the DM12 element and stress-based elements.

As the strain energy for kinematically admissible and statically admissible solutions is known, it is possible to evaluate the upper bound for the relative error of the approximate solution by Synge’s method [24,30]. It is pictured in Figure 7 for both plate thicknesses.

The errors calculated on the basis of both stress-based elements and the displacement-based DM22 element are presented on the left side of the figure, whereas results involving the DM12 element are shown on the right side. The errors evaluated on the basis of Synge’s method depend on the gap between the values of the strain energy obtained by both kinematically and statically admissible methods. Therefore, values of the errors that include results of DM12 elements are much larger than those obtained using the DM22 element. The reason for that is a much slower convergence rate of the DM12 element (visible in Figure 6). In the case of the coarsest mesh, the errors for the DM12 element are larger than 40% for a thinner plate and almost 24% for a thicker plate, and they decrease to values of 6.1–6.9% for the finest mesh. When the DM22 element is taken as a counterpart of the stress-based elements, the values of the errors decrease to the level of 2.4–3.7% for the mesh of size $h_{\max}=a/4$ and are around 0.2% for the mesh of size $h_{\max}=a/16$. The error values are close to each other regardless of the type of the stress-based element used in calculations as they both give similar energy values. Therefore, lines representing errors computed using HCT and BFS elements (with respect to the same plate thickness and the same displacement-based element) overlap each other.

**Figure 8:**
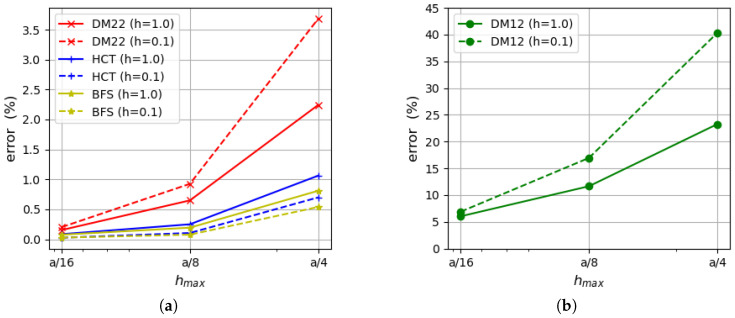
Relative error of approximate solution calculated on the basis of Equation (65): (**a**) for the DM22, BFS and HCT elements; (**b**) for the DM12 element.

For a comparison of results, values of the relative error of approximate solutions also calculated as

(65) $$\begin{array}{c} \Delta =\sqrt{\frac{\left|E_{a}-E_{\mathrm{app}}\right|}{E_{a}}}\times 100\% \end{array}$$

are shown in Figure 8. $E_{a}$ is the analytically found value of the strain energy [35] and $E_{\mathrm{app}}$ means the approximate value of the energy computed by the kinematically or statically admissible method. Figure 8 confirms the previously drawn conclusions that the equilibrium-based elements provide more precise solutions, even in comparison with the DM22 element. The most accurate results are obtained by the BFS element, but the HCT element provides us with similarly good approximation as the difference between these two elements is negligible. All the errors obtained for the equilibrium FEM are lower than 1.1% for the mesh of size $h_{\max}=a/4$ and are smaller than 0.09% when a mesh with maximum element size $h_{\max}=a/16$ is used. It is an excellent result in comparison with the displacement-based elements, as for the DM22 triangle, the errors are more than twice as large for the coarsest mesh and 1.81–2.02 times larger for the densest mesh; meanwhile, for the DM12 element, the error is in the range of 23–40% for the coarsest mesh and decreases to the level of 6% within mesh refinement. All the errors calculated by the described elements reach larger values if the thinner plate is analysed.

**Figure 9:**
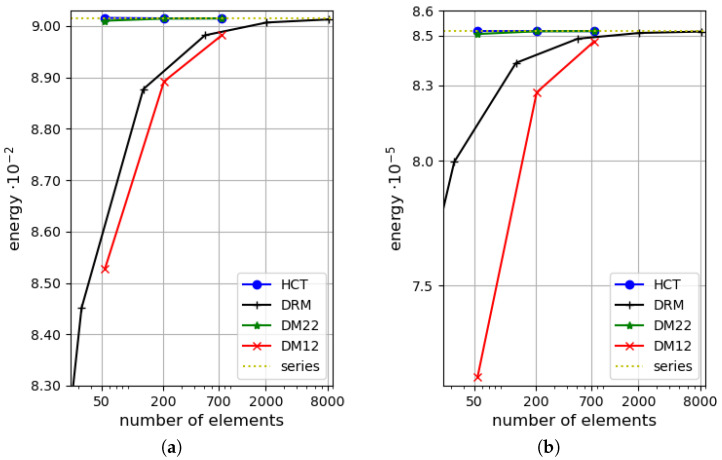
Efficiency comparison for triangular elements HCT, DM12, DM22 and DRM (discrete Reissner–Mindlin [35]): (**a**) for h=1.0; (**b**) for h=0.1. The simply supported, rectangular plate.

The efficiency of the proposed triangular, stress-based HCT element and the displacement-based elements described in Section 4 is compared with the efficiency of the triangular DRM (discrete Reissner–Mindlin) element proposed by Zienkiewicz et al. [35]. This element has 21 degrees of freedom and is a generalisation of the DKT element (discrete Kirchhoff triangle; see references in [35] for details). The strain energy–number relation of mesh elements is shown in Figure 9 for both values of the plate thickness. Dependency on the elements number is illustrated as in [35].

The diagrams in the figure reveal a distinct advantage of the stress-based HCT element over the DRM element. Similarly, the DM22 element is more efficient than the DRM one.

The same calculations are performed for a square plate with clamped boundaries. The data and element grids used in the computations are the same as above.

**Figure 10:**
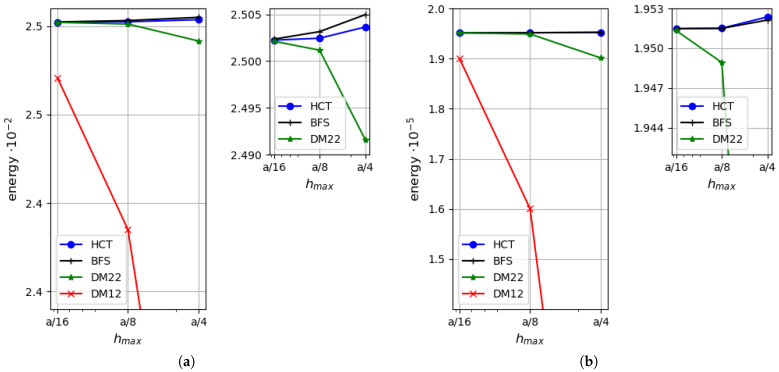
Strain energy depending on the maximum size of plate elements: (**a**) for h=1.0; (**b**) for h=0.1.

Figure 10 presents a comparison of the strain energy values computed using four types of elements (DM12, DM22, HCT and BFS) for two values of plate thickness (h=1.0 on the left and h=0.1 on the right). In both cases, all the results converge monotonically to one value. The displacement-based elements give the lower bound, whereas the stress-based elements give the upper bound for the strain energy. As in the previously analysed example, the convergence rate is visibly faster when the equilibrium approach is used.

**Figure 11:**
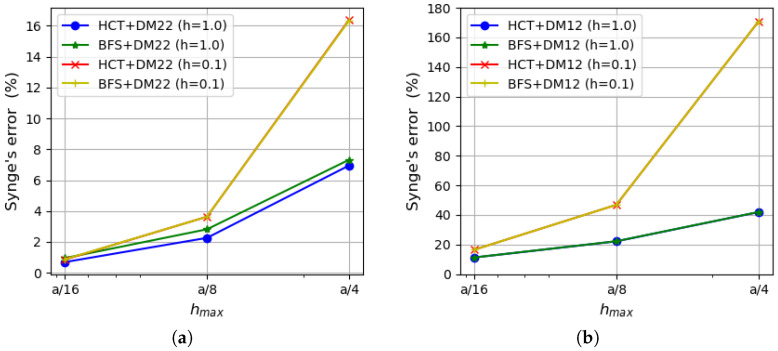
Synge’s error depending on the maximum size of plate elements: (**a**) using the DM22 element and stress-based elements; (**b**) using the DM12 element and stress-based elements.

An analysis of Figure 11, which presents the errors calculated by Synge’s method, confirms that larger values of the errors are obtained in the case of thinner plates. The error values that involve the DM22 element in conjunction with the stress-based elements are satisfactorily small and less than 1% for the finest mesh. The errors evaluated on the basis of the DM12 element are much larger due to the fact that this element is not as accurate as the three others.

### 5.2. Uniformly Loaded Simply Supported Circular Plate

The next example is a circular plate with radius *R* under uniform loading *q*. “Hard” simply supported boundary conditions are considered. The material properties are E=10.92 and ν=0.3, which gives a plate stiffness $Eh^{3}/\left(12\left(1-\nu^{2}\right)\right)$ of $h^{3}$. The radius *R* is set to 5. Plate thickness is considered in two variants: thin h=0.1 (R/h=50) and thick h=1.0 (R/h=5). The intensity of the uniform loading *q* is 1.0. The material and loading data as well as plate dimensions are set as in Ref. [35].

**Figure 12:**
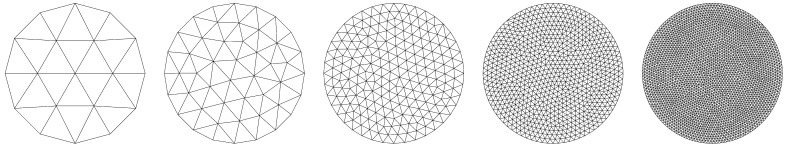
Element grids for a circular plate.

Five various element grids shown in Figure 12 are examined in the case of the equilibrium version of the HCT element and two displacement-based elements DM12 and DM22.

It is assumed that the element edges are straight; then, the plate boundary is discretised as a polygon. To conduct the convergence analysis in a reasonable way, it is important to keep the total loading or plate area the same for all element grids. This is achieved by properly controlling the distance between boundary nodes and the plate centre.

To keep the approximate stress-based solution as close as possible to the exact axi-symmetric solution, the vertical corner forces that can appear in the stress-based model are eliminated in the manner described in Section 3.1.

In the displacement-based model, the Kirchhoff condition (28) cannot be applied for the boundary directly because the rotations in two different directions would be set at corner nodes. This would mean that the plate boundary is clamped. It is assumed in the computations that for any boundary node, the rotation angle in the plane perpendicular to the line connecting the node and the plate centre is equal to zero. Obviously, this assumption somewhat violates the kinematic admissibility of the displacement field.

**Figure 13:**
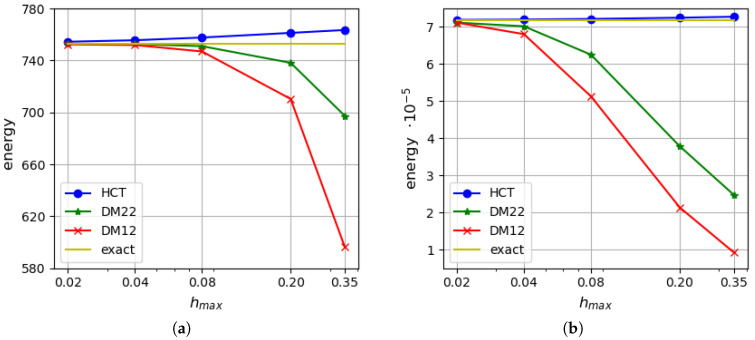
Strain energy: (**a**) for h=1.0; (**b**) for h=0.1.

Figure 13 presents a comparison of the strain energy values calculated for the analysed plate depending on the maximum size of plate elements. The results obtained for thickness h=1.0 and h=0.1 are presented on the left and right diagrams, respectively. It is visible that the stress-based method performs a precise approximation, even for the coarse element mesh. The stress-based HCT element shows much faster convergence in comparison with both displacement-based elements.

**Figure 14:**
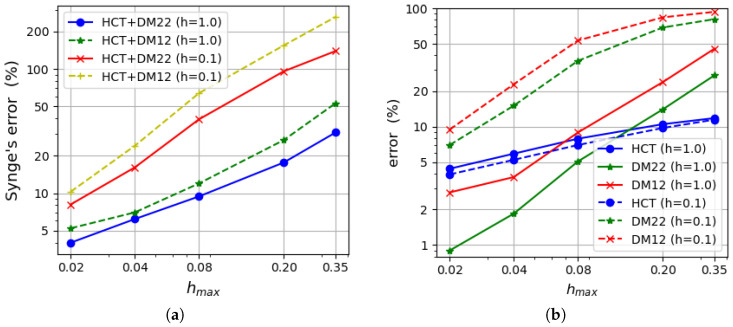
(**a**) Synge’s error estimation. (**b**) Actual error (Equation (65)).

Figure 14 displays the Synge’s error estimation compared with the actual error of the approximate solution calculated according to Equation (65). Synge’s method overestimates the actual error value (especially in the case of thinner plate). This is a result of the lower accuracy of the displacement-based elements. When the convergence rate of two dual methods is similar, Synge’s method yields values of errors only slightly larger than the actual errors of individual elements.

**Figure 15:**
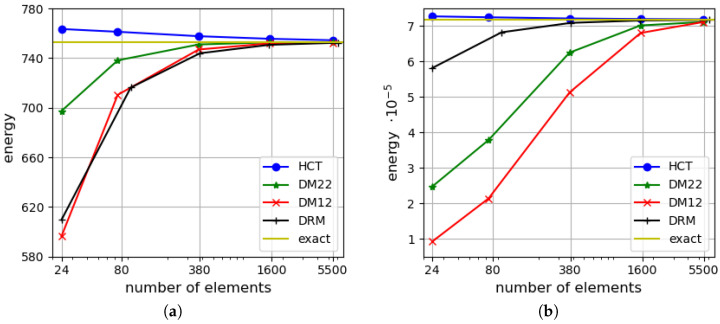
Efficiency comparison for triangular elements HCT, DM12, DM22 and DRM (discrete Reissner–Mindlin [35]): (**a**) for h=1.0; (**b**) for h=0.1. The simply supported, circular plate.

Again, as in the previous subsection, an efficiency comparison for triangular elements HCT, DM22, DM12 and DRM [35] is performed. The dependency of the strain energy on the number of grid elements is depicted in Figure 15 for two values of the thickness of the plate. Similarly to the case of the rectangular plate analysed above, it is clearly visible that the proposed stress-based HCT element is much more efficient than the DRM element [35].

It is observed that the accuracy of the displacement-based elements DM22 and DM12 is deteriorated for the case of the thinner plate (h=0.1), presumably due to difficulties related to the approximation of a curved boundary by a set of straight segments mentioned above.

### 5.3. Uniformly Loaded Rectangular Plate

**Figure 16:**
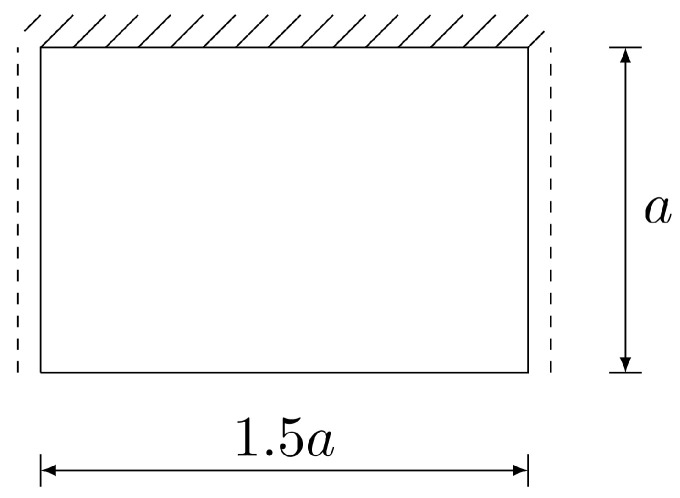
Rectangular plate loaded uniformly.

**Figure 17:**
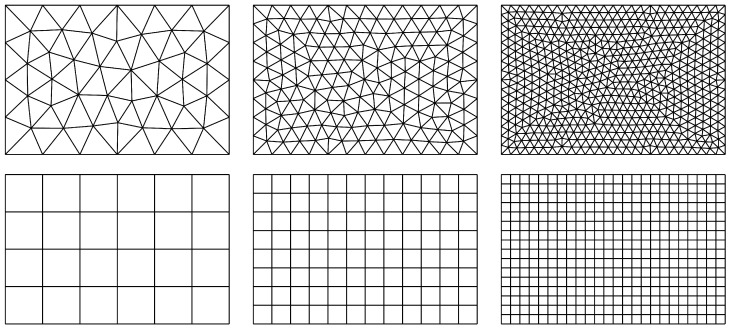
Computational grids for HCT, DM12 and DM22 elements (upper diagrams) and for the BFS element (lower diagrams).

The last example is a rectangular plate of size a×1.5a (with a=1m) subjected to uniformly distributed loading $q=10^{3}\;N/m^{2}$. Two opposite edges are simply supported, one edge is clamped and one is free. The plate with its dimensions is shown in Figure 16. Three element grids are utilised, with the maximum element size being taken as a/4, a/8 and a/16. All the grids used in the calculations are presented in Figure 17. The results obtained by use of the equilibrium HCT and BFS elements are compared with the results gained by the displacement-based DM12 and DM22 elements.

The plate is considered isotropic and homogeneous with Young’s modulus E=200GPa and Poisson’s ratio ν=0.3.

**Figure 18:**
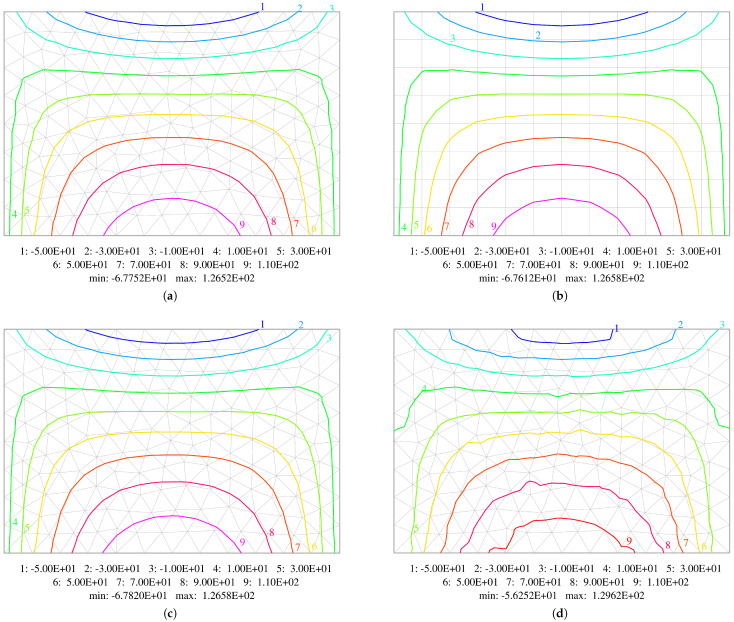
Comparison of contour plots of bending moments $M_{xx}$. Results obtained using: (**a**) HCT element; (**b**) BFS element; (**c**) DM22 element; (**d**) DM12 element.

**Figure 19:**
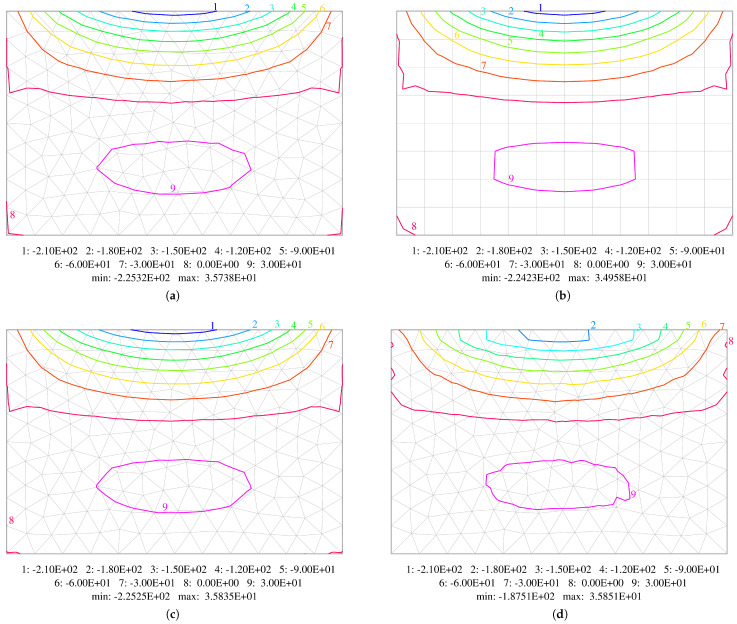
Comparison of contour plots of bending moments $M_{yy}$. Results obtained using: (**a**) HCT element; (**b**) BFS element; (**c**) DM22 element; (**d**) DM12 element.

**Figure 20:**
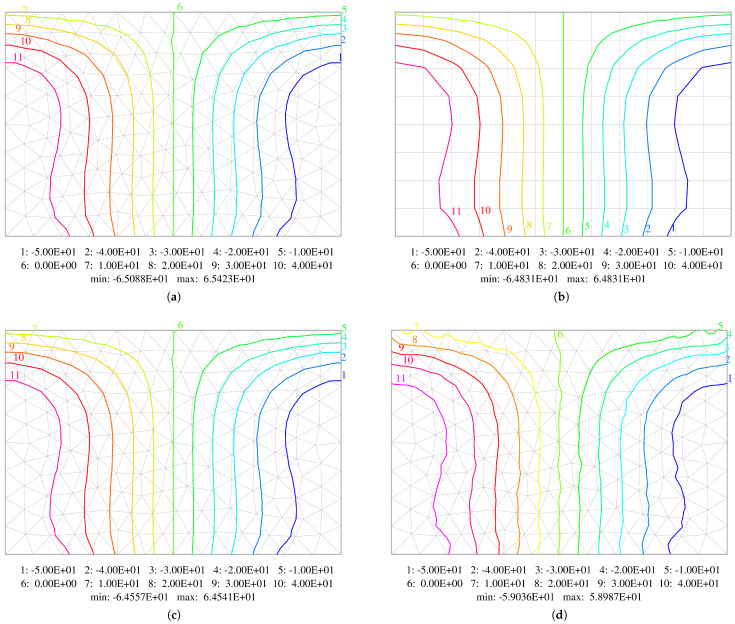
Comparison of contour plots of twisting moments $M_{xy}$. Results obtained using: (**a**) HCT element; (**b**) BFS element; (**c**) DM22 element; (**d**) DM12 element.

A comparison of the isolines of bending moments $M_{xx}$ and $M_{yy}$, as well as twisting moments $M_{xy}$ obtained using middle mesh for plate thickness h=0.05 m, is shown in Figure 18, Figure 19 and Figure 20. A thinner plate is chosen as the errors are bigger in this case. Diagrams concerning all the elements used in the calculations are displayed. They confirm that both methods (displacement-based and stress-based) yield very similar results. All the contour plots are symmetric and exhibit resemblance. The plots for HCT and DM22 elements are almost identical. The BFS element gives very similar results, which is very satisfactory concerning a different mesh subdivision pattern. The largest difference is observed in the case of the DM12 element, which has the lowest accuracy. The contour plots are not as smooth as in the case of the three other elements. To construct isoline plots, first the nodal values of a specific variable are calculated for each element using the moving weighted least squares method [36] (MWLS). Then, global nodal values are calculated by simple averaging.

A comparison of maximum and minimum values of bending moments $M_{xx}$ obtained at nodes (after post-processing) is presented in Table 2 and Table 3, respectively, taking into account all the element types and grids. An agreement in three significant digits is seen for grids of size a/8 and four digits for the densest mesh ($h_{\max}=a/16$) in the case of HCT, BFS and DM22 elements for two values of plate thickness when maximum values are compared. When a comparison of minimum values is performed, two significant digits are the same for $h_{\max}=a/8$ and $h_{\max}=a/16$ (except for the mesh of middle density for the plate of thickness h=0.3 m using the BFS element). Naturally, the DM12 element does not offer such a good agreement.

**Figure 21:**
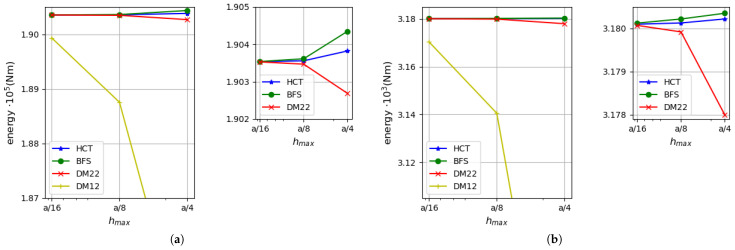
Strain energy: (**a**) for h=0.3 m; (**b**) for h=0.05 m.

Convergence of the results is verified for thin (h=0.05 m) and moderately thick (h=0.3 m) plates. Figure 21 presents a comparison of strain energy values calculated using all the mentioned elements. The left diagrams present the energy computed in the case of a thicker plate (h=0.3 m), while the right ones show results for a thinner plate (h=0.05 m). The results for both plate thicknesses confirm that the upper bound for the strain energy is found if the stress-based method is used and the lower bound is provided by the displacement-based analysis. The convergence rate of the equilibrium-based elements is higher than their displacement-based counterparts. As expected, the DM12 element is characterised by the slowest convergence rate for both plate thickness values. The DM22 element provides a comparable convergence rate for thicker plates as the BFS rectangle, but its performance is not so high when thinner plates are analysed. The HCT triangle is the most precise in both the cases.

**Figure 22:**
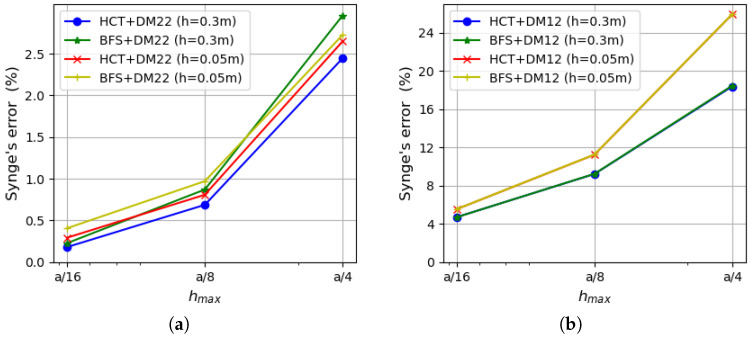
Synge’s error: (**a**) for equilibrium-based elements with the DM22 element; (**b**) for equilibrium-based elements with the DM12 element.

The relative error of the approximate solution is calculated by means of Synge’s method [24,30] for all combinations of the equilibrium-based and displacement-based results. Figure 22 presents how the error depends on the maximum element size.

It is presented in the form of two diagrams in order to maintain clarity. The left diagram refers to errors computed for both plate thicknesses using results for the DM22 element as the kinematically admissible solution, whereas the right diagram uses the DM12 element instead. Synge’s method includes the gap between values of the strain energy obtained by dual solutions. The DM12 element uses shape functions of a lower degree than the DM22 element and is therefore less accurate. This is the reason why values of the error presented on the right side of Figure 22 are much larger. An acceptable level of error is obtained for the densest mesh as it does not exceed 4%. The errors calculated using DM22 and equilibrium elements are close to 2% in the case of the coarsest computational grid and decrease to less than 0.3% for the densest mesh.

## 6. Concluding Remarks

Two stress-based elements of class $C^{1}$ are successfully implemented in the Mindlin plate analysis. Using Southwell’s stress functions enables one to obtain statically admissible field of stress resultants (bending and twisting moments and shearing forces) inside the plate area. The simpler case of hard (2D) boundary conditions is analysed; the solution of the soft case of boundary conditions is a more difficult task. The proposed multi-point-constraints edge elements enforce hard boundary conditions along simply supported and free edges. In the case of a clamped edge, enforcing the boundary conditions is not needed. The method yields trustworthy results for plates of a wide range of thicknesses.

The computational results obtained by the stress-based HCT and BFS elements are compared with those acquired by two displacement-based DM12 and DM22 elements. A higher speed of convergence of the approximate solution is observed for HCT (24 degrees of freedom) and BFS (32 degrees of freedom) than for the more accurate (comparing to DM12) displacement-based DM22 element (22 degrees of freedom).

A much higher efficiency of the stress-based HCT element is also shown compared with the well known direct Reissner–Mindlin (DRM) element with 21 degrees of freedom from the literature.

The upper and lower bounds for the strain energy are found by the two dual approaches to the FEM. The a posteriori error estimation is performed using Synge’s method. A comparison between the approximate solutions and analytic solutions available for some problems shows very good agreement between them.

A future aim of the research is the extension of the stress-based approach in the case of the soft (3D) boundary conditions. Another future direction of the study is a generalisation of the method on the elastic–plastic analysis, which will allow one to find the lower value of the limit loading.

## Figures and Tables

**Table 1:** Two kinds of boundary conditions for the plate bending problem.

	Hard Conditions	Soft Conditions
Clamped edge:	w=0, $\theta_{n}=0$, $\theta_{s}=0$	w=0, $\theta_{n}=0$, $M_{ns}=0$
Simply supported edge:	w=0, $M_{nn}=0$, $\theta_{s}=0$	w=0, $M_{nn}=0$, $M_{ns}=0$
Free edge:	$Q_{K}=\bar{Q}$, $M_{nn}=\bar{M}$, $w_{,s}-\theta_{s}=0$	$Q_{n}=0$, $M_{nn}=0$, $M_{ns}=0$

**Table 2:** Comparison of maximum values of bending moments $M_{xx}$ [N].

Mesh	h = 0.3 m				h = 0.05 m			
$h_{\max}$	**HCT**	**BFS**	**DM22**	**DM12**	**HCT**	**BFS**	**DM22**	**DM12**
a/4	1.4209	1.4187	1.4213	1.4547	1.2753	1.2767	1.2800	1.2967
a/8	1.4139	1.4134	1.4142	1.4525	1.2652	1.2658	1.2658	1.2962
a/16	1.4126	1.4124	1.4126	1.4371	1.2626	1.2627	1.2620	1.2833

**Table 3:** Comparison of minimum values of bending moments $M_{xx}$ [N].

Mesh	h = 0.3 m				h = 0.05 m			
$h_{\max}$	**HCT**	**BFS**	**DM22**	**DM12**	**HCT**	**BFS**	**DM22**	**DM12**
a/4	−5.7174	−5.5222	−5.6741	−3.9544	−6.7870	−6.7078	−6.8105	−4.2447
a/8	−5.8558	−5.7913	−5.8529	−4.9958	−6.7752	−6.7612	−6.7820	−5.6252
a/16	−5.8858	−5.8718	−5.8962	−5.4176	−6.7735	−6.7685	−6.7650	−6.1566

## Data Availability

The original contributions presented in this study are included in the article. Further inquiries can be directed to the corresponding author.
